# Malnutrition and inflammation in acute kidney injury due to earthquake-related crush syndrome

**DOI:** 10.1186/1471-2369-11-4

**Published:** 2010-03-27

**Authors:** Gui-Sen Li, Xiu-Ling Chen, Yuan Zhang, Qiang He, Fang Wang, Da-Qing Hong, Ping Zhang, Lei Pu, Yue Zhang, Xiu-Chuan Yang, Li Wang

**Affiliations:** 1 Department of Nephrology, Institute of Nephrology, Sichuan Academy of Medical Sciences & Sichuan Provincial People's Hospital, Chengdu, Sichuan, China

## Abstract

**Background:**

Malnutrition and inflammation are common and serious complications in patients with acute kidney injury (AKI). However, the profile of these complications in patients with AKI caused by crush syndrome (CS) remains unclear. This study describes the clinical characteristics of malnutrition and inflammation in patients with AKI and CS due to the Wenchuan earthquake.

**Methods:**

One thousand and twelve victims and eighteen healthy adults were recruited to the study. They were divided into five groups: Group A was composed of victims without CS and AKI (904 cases); Group B was composed of patients with CS and AKI who haven't received renal replacement therapy (RRT) (57 cases); and Group C was composed of patients with CS and AKI receiving RRT (25 cases); Group D was composed of earthquake victims with AKI but without CS (26 cases); and Group E was composed of 18 healthy adult controls. The C-reactive protein (CRP), prealbumin, transferrin, interleukin-6 and TNF-α were measured and compared between Group E and 18 patients from Group C.

**Results:**

The results indicate that participants in Group C had the highest level of serum creatinine, blood urea nitrogen and uric acid. Approximately 92% of patients with CS who had RRT were suffering from hypoalbuminemia. The interleukin-6 and CRP levels were significantly higher in patients with CS AKI receiving RRT than in the control group. Patients in Group C received the highest dosages of albumin, plasma or red blood cell transfusions. One patient in Group C died during treatment.

**Conclusions:**

Malnutrition and inflammation was common in patients with earthquake-related CS and had a negative impact on the prognosis of these subjects. The results of this study indicate that the use of RRT, intensive nutritional supplementation and transfusion alleviated the degree of malnutrition and inflammation in hemodialysis patients with crush syndrome.

## Background

Acute renal failure, which is now referred to as acute kidney injury (AKI) [1], is a significant clinical problem that has an independent and major negative impact on patient outcomes [1-3]. Up to 5% of patients with AKI in intensive care units (ICU) may require renal replacement therapy (RRT) [3], and AKI has been demonstrated to be a major independent risk factor for mortality in patients with critical illnesses [4,5]. In specific cases of patients with AKI, nutritional status has been found to be closely related to the rates of comorbidities and mortality [6-10]. A recent report also demonstrated that serum prealbumin levels of less than 11 mg/dL were associated independently with a higher risk of death [11]. Nutritional depletion and protein consumption has also been found to influence the effectiveness of treatment and worsen the prognosis of patients with AKI [7,9,10].

Until now, the characteristics of malnutrition in patients with AKI caused by crush syndrome (CS) have remained unclear. Since CS accelerates catabolism, which would theoretically aggravate malnutrition, this is an important issue. AKI and CS caused by earthquakes usually result in far worse clinical outcomes because of the severe complications that typically ensue [12-14]. Previous reports reveal that CS and subsequent AKI is the main cause of death following an earthquake [12,13]. Furthermore, AKI results not only in fundamental alterations to metabolism, but also in the activation of a pro-oxidative and proinflammatory state [7,15]. Many inflammatory biomarkers are activated in patients with AKI and these would further aggravate the malnutrition of such patients [7,15]. It is therefore vital to determine the characteristics of malnutrition and inflammation in patients with AKI that resulted from earthquake-related CS.

In this article, the characteristics of malnutrition and inflammation in participants with CS and AKI following the Wenchuan earthquake are summarized. The effects of RRT on the status malnutrition and inflammation of participants are also investigated.

## Methods

During the Wenchuan earthquake, the Sichuan Academy of Medical Sciences and Sichuan Provincial People's Hospital was one of the closest hospitals to the epicenter and, as a result, many patients with injuries due to trauma were admitted to the hospital immediately after the earthquake.

One thousand and twelve victims and eighteen healthy adults were recruited to the study. They were divided into five groups: Group A was composed of victims without CS and AKI (904 cases); Group B was composed of patients with CS and AKI who haven't received renal replacement therapy (RRT) (57 cases); and Group C was composed of patients with CS and AKI receiving RRT (25 cases); Group D was composed of earthquake victims with AKI but without CS (26 cases); and Group E was composed of 18 healthy adult controls.

Serum total protein and serum albumin levels were compared between these three groups during the first three days of their admission. The serum C-reactive protein (CRP), prealbumin, and transferrin levels were measured in participants in Group C and Group E. Interleukin 6 (IL-6) (Wuhan Boster Biological Technology, China) and tumor necrosis factor-α (TNF-α) (Wuhan Boster Biological Technology, China) levels were tested by enzyme-linked immunosorbent assay (ELISA) methods in accordance with the specifications of the manufacturers in Group E and in 18 participants from Group C who received RRT and were followed for more than 14 days. The serum samples of these patients were collected on the first day and the forteenth day of RRT.

The clinical parameters (included blood pressure, hemoglobin, blood urea nitrogen, and serum creatinine levels, total serum protein, serum albumin, serum electrolytes, etc. All of them were detected on the admission day) of these patients were also compared. We also recorded the dosages of infused albumin, plasma, or red blood cell in three groups. All of the patients with CS and AKI were given nutritional supports by intravenous infusion and/or oral supplements. The modalities of RRT in Group C included intermittent hemodialysis (IHD) (7 cases), continuous veno-venous hemofiltration (CVVH) (11 cases), and modality from IHD to CVVH (8 cases).

For the purpose of this study, CS was diagnosed on the basis of the presence of swollen limbs and history of limb compression according to the criteria described previously [16,17]. AKI was defined as an abrupt (within 48 hours) reduction in kidney function, which is defined currently as an absolute increase in serum creatinine of more than or equal to 0.3 mg/dl (≥26.4 μmol/l), a percentage increase in serum creatinine of more than or equal to 50% (1.5-fold from baseline), or a reduction in urine output (documented oliguria of less than 0.5 ml/kg per hour for more than six hours) [1]. AKI was classified into three stages following the AKIN criteria [1].

These clinical data were collated and checked by another physician. Statistical analysis was conducted using SPSS version 13.0 (SSPS Inc, Chicago, IL, USA).

The protocol for this study was approved by medical ethics committee of the Sichuan Academy of Medical Sciences and Sichuan Provincial People's Hospital, and informed written consent for the study was obtained from all of the patients with RRT or healthy adults. For the other patients without AKI, this study was an audit of historical data without any interventions, and therefore, the chairman of the hospital medical ethics committee confirmed that formal ethical approval was not required.

## Results

### The clinical characteristics of the victims with or without acute kidney injury

The clinical characteristics among Group A, B and C were compared. The results revealed that the participants with CS and AKI had significantly increased the levels of BUN, serum creatinine, plasma glucose, uric acid, serum potassium, serum phosphorus, aspartate aminotransferase (AST), serum lactate dehydrogenase (LDH) and creatinine kinase (CK). On the other hand, the hemoglobin, bicarbonate, and calcium levels had decreased significantly. All of these abnormalities were more prominent in CS and AKI patients with RRT (Table 1). One participant in Group C died because of severe sepsis.

**Table 1:** The characteristics of patients with crush syndrome and acute kidney injury.

	Group A *n *= 904	Group B *n *= 57	Group C *n *= 25
Age (mean years ± SD)	46.2 ± 24.4	55.6 ± 22.2*	36.1 ± 16.4*^#^
Male (%)	50.3	45.6	52.0
Mean arterial pressure (mmHg)	90.9 ± 12.3	92.2 ± 13.7	89.8 ± 15.1
White blood cells (×10^9^/L)	8.01 ± 3.81	9.25 ± 4.36	11.12 ± 6.00*
Hemoglobin (g/L)	107.8 ± 26.5	103.7 ± 30.6	87.2 ± 31.4**^#^
Platelet (×10^9^/L)	175.3 ± 95.2	128.4 ± 45.7**	104.8 ± 69.5**
Blood urea nitrogen (mmol/L)	6.05 ± 3.83	12.19 ± 6.65**	22.90 ± 10.62**^##^
Serum creatinine (μmol/L)	80.4 ± 63.6	163.2 ± 73.6**	419.1 ± 141.5**^##^
Glucose (mmol/L)	5.86 ± 2.36	7.07 ± 4.10**	7.33 ± 4.66**
Uric acid (μmol/L	233.9 ± 113.5	348.0 ± 234.5*	486.1 ± 230.2**^##^
Potassium (mmol/L)	3.93 ± 0. 51	4.12 ± 0.80	5.80 ± 1.27**^##^
HCO_3 _^-^(mmol/L)	25.4 ± 3.8	24.0 ± 5.3*	20.1 ± 5.9**^##^
Calcium (mmol/L)	2.20 ± 0.22	2.08 ± 0.32**	1.71 ± 0.25**^##^
Phosphorus (mmol/L)	1.13 ± 0.42	1.31 ± 0.56	1.74 ± 0.76**
Total protein (g/L)	60.8 ± 9.3	57.8 ± 11.4	43.5 ± 11.5**^##^
Albumin (g/L)	34.6 ± 6.5	32.3 ± 7.0	22.0 ± 8.5**^##^
Aspartate aminotransferase (U/L)	38(28, 59)	52(35, 309)*	768(336, 1213)**^##^
Serum lactate dehydrogenase (U/L)	307(214, 541)	615(318, 1157)*	5451(2532, 9964)**^##^
Creatinkinase (U/L)	166 (75, 731)	216 (116,1017)	18636(7585, 23852)**^##^

Group A, victims without crush syndrome (CS) and acute kidney injury (AKI); Group B, patients with CS and AKI haven't received renal replacement therapy (RRT); Group C, patients with CS and AKI have received RRT. Compared to Group A, *:p < 0.05, **:p < 0.01; Compared to Group B, ^#^:p < 0.05, ^##^: p < 0.01.

The serum total protein and albumin levels were found to have significantly decreased in participants who were treated by RRT. The mean serum albumin was only 22.0 ± 8.5 g/L (range: 8.8-49.3 g/L) in Group C participants. In Group B participants, 57.9% (33/57) patients had an albumin level of less than 35 g/L. The proportion was 92.0% (23/25). Moreover, 16 patients (64.0%) had an albumin level of less than 25 g/L in the participants of Group C.

### The clinical characteristics of AKI patients with or without crush syndrome

To clarify the clnical manifetations of AKI patients with CS, We compared the renal function, plasma total protein, and albumin levels between participants in Group B and in Group D. The participants with AKI and CS were older and had significantly higher levels of BUN, serum creatinine, serum potassium. However, the serum calcium, total plasma protein, and albumin levels were significantly lower in these patients than those without CS (Table 2).

**Table 2:** The renal function and plasma protein in AKI patients with or without CS.

	Group B *N *= 57	Group D *N *= 26	*P *value
Age	41.5 ± 19.0	67.4 ± 18.9	1.6 × 10^-7^
Male(%)	50.0	42.3	0.516
Hemoglobin (g/L)	98.5 ± 33.7	98.7 ± 26.6	0.979
Blood urea nitrogen (mmol/L)	17.33 ± 10.27	11.37 ± 5.46	1.0 × 10^-3^
Serum creatinine (μmol/L	280.0 ± 169.2	157.7 ± 57.9	6.8 × 10^-6^
Uric acid (μmol/L	391.1 ± 248.8	393.1 ± 224.3	0.975
Potassium (mmol/L)	5.04 ± 1.26	3.79 ± 0. 59	4.56 × 10^-8^
Calcium (mmol/L)	1.88 ± 0.30	2.10 ± 0.38	0.015
Phosphorus (mmol/L)	1.52 ± 0.68	1.33 ± 0.62	0.283
Total protein(g/L)	50.6 ± 12.8	60.6 ± 11.2	1.3 × 10^-3^
Albumin (g/L)	27.1 ± 9.1	33.6 ± 6.2	3.7 × 10^-4^

Group B, AKI patients with CS; Group D, AKI patients without CS

### The inflammation markers and the effect of RRT

The markers of inflammation (CRP, prealbumin, transferrin, IL-6, and TNF-α) were detected in 18 healthy controls and in 18 participants from Group C who received RRT and were followed for more than 14 days. The participants with CS and AKI had significantly higher serum levels of IL-6 and CRP than the normal controls. The serum levels of prealbumin and transferrin decreased significantly in these patients (Table 3). After 14 days of RRT, the serum levels of IL-6 and TNF-α were 756.5 ± 288.0 ng/ml and 40.8 ± 16.0 ng/ml, respectively, and these did not change significantly from the pre-treatment levels.

**Table 3:** The inflammatory markers in AKI patients treated by RRT and in controls.

	Group E *N *= 18	**Group C**^**a**^ *N *= 18
Age	42.8 ± 15.3	42.1 ± 20.3
Male (%)	44.4	55.6
CRP(mg/L)	5.1 ± 2.6	107.7 ± 61.2**
Prealbumin (mg/L)	368.2 ± 42.5	200.0 ± 58.8**
Transferrin (ng/L)	2.76 ± 0.65	1.02 ± 0.48*
IL-6 (ng/mL)	334.3 ± 117.2	882.7 ± 306.8**
TNF-α (ng/ml)	39.5 ± 10.1	42.3 ± 16. 9

^a^18 participants from Group C who received RRT and were followed for more than 14 days.*p < 0.05, **p < 0.01.

### RRT influenced markers of inflammation and nutrition

During the period of treatment, providing transfusions of albumin, plasma, or red blood cells was one of the most effective methods of treatment. The supplements used in the different groups are displayed in Table 4. Participants in Group C received the most dosages of albumin, plasma and red blood cells.

**Table 4:** The dosages of plasma, red blood cell or albumin that were supplemented in the different groups.

	Group A *N *= 904	Group B *N *= 57	Group C *N *= 25
Plasma (ml)	0(0,5950) [31.7]^a^	0(0, 1200) [25.9]	1275.0(0,7900) [2181.8]*
Red blood cell (U)	0 (0, 45) [0.56]	0(0,8) [0.42]	3.0(0,24) [5.32]*
20% Albumin (ml)	0 (0, 150) [0.3]	0(0,200) [6.4]	0(0, 550) [101.1]*

^a ^These values were expressed as median (minimum, maximum) [mean]. * The *p *value was less than 10^-5^.

## Discussion

It has been reported that malnutrition is common in patients with AKI and that nutritional status is closely related to the outcomes of these patients [6-10]. In particular, malnutrition has often been found to result in increased the mortality among these patients [6-10]. A previous report revealed that the mortality in acute renal failure patients with severe malnutrition was significantly higher than in those with normal nutritional status (80/129 vs. 24/130) [6]. However, the nutritional problems in patients with CS and AKI has remained unclear and the only prior description of this problem did not include detailed data [18]. This study reported the clinical characteristics of malnutrition and inflammation in patients with earthquake-related CS and AKI. The results demonstrated that malnutrition was a common complication in patients with CS and AKI and was also typically more severe in patients with CS and AKI who received RRT.

Recently, the International Society of Renal Nutrition and Metabolism (ISRNM) convened an expert panel to review and develop standard terminologies and definitions related to wasting, cachexia, malnutrition, and inflammation in chronic kidney disease and AKI. The ISRNM expert panel recommends the term 'protein-energy wasting' for loss of body protein mass and fuel reserves [19]. Decreased serum albumin is the main diagnostic indicator of malnutrition in patients with AKI. However, nutritional status evaluation in this clinical condition is difficult because most of the traditional nutritional tools (e.g. body weight and body mass index, anthropometric measurements, and serum protein levels) are often misleading, owing to the presence of acute illness and/or of alterations in body water distribution and external fluid balance derangements [10,19]. In this study, it was found that the serum total protein and albumin levels decreased significantly in CS and AKI participants without RRT and was much lower still in CS and AKI participants with RRT. In the participants with CS and AKI who were treated by RRT, the mean level of serum albumin was only 22.0 g/L and 92% of these participants had decreased serum albumin of less than 35 g/L. These participants also had higher levels of AST, LDH, CK, and creatinine. Compared to those participants with AKI but without CS, the participants with both CS and AKI had lower levels of total protein and albumin. These results indicate that both AKI and CS contribute to the malnutrition status of earthquake victims.

Multiple factors could explain the observed malnutrition in these participants. These include anorexia, impaired protein metabolism and transport, oxidative stress, production of nonspecific inflammatory cytokines, resistance to insulin, volume overload, metabolic acidosis, nutrient losses through the hemodiafilter, and patient comorbidities [7,10,19]. This indicates that AKI, which results in the loss of lean body mass, may not be primarily related to reduced nutrient intake. In participants with both CS and AKI, the malnutrition resulted from not only above-mentioned causes, but also from exudation and bleeding related to injuries sustained during the earthquake. These participants also demonstrated prominent inflammation, which may have contributed to their malnutrition. In our study, the serum levels of IL-6 and CRP increased significantly and the serum levels of prealbumin and transferrin decreased significantly in the CS and AKI participants who were treated with RRT. Furthermore, the IL-6 did not decrease after RRT for 14 days. This indicates that the mechanisms of inflammation continued to exacerbate the malnutrition of these patients by accelerating catabolism and inhibiting anabolism. The data presented herein demonstrates that the inflammatory process in the participants with CS and AKI was severe.

Therefore, in patients with AKI, a close investigation between nutritional support and RRT is required, especially when highly efficient RRTs, such as CVVH, or daily prolonged intermittent RRT, such as sustained low-efficiency dialysis (SLED), are used [7,10,19,20]. Adequate nutritional support is necessary to maintain protein stores and to correct pre-existing or disease-related deficits in the lean body mass. CS patients with AKI following an earthquake, especially those treated with RRT, are at more risk of nutritional depletion than other critically ill patients. The results presented here indicate that patients with CS and AKI require specific nutritional support involving the supplementation of albumin, plasma and red blood cells, all of which are main treatments for patients with CS and AKI following an earthquake. In our study, participants with CS and AKI who were treated by RRT received the highest dosages of albumin, plasma and red blood cells. Only one patient died because of severe sepsis. These results suggest that nutritional support is one of the most important treatments for the patients with AKI resulting from CS, especially in those patients who were treated by RRT.

## Conclusions

Malnutrition and inflammation was common in patients with earthquake-related crush syndrome and acute kidney injury. These patients had more obvious hypoproteinemia, hypoalbuminemia, and anemia. They also had higher levels of inflammation markers and needed more nutritional supplements. The results of this study indicate that the use of RRT, intensive nutritional supplementation and transfusion could alleviate the degree of malnutrition and inflammation in patients with crush syndrome and acute kidney injury after massive earthquake.

## Competing interests

The authors declare that they have no competing interests.

## Authors' contributions

LGS and CXL collected and processed data, helped with the design of the study and drafted the manuscript. ZY, HQ, and WF collected the data. HDQ, ZP, PL and ZY analyzed the inflammatory markers in the serum samples. YXC participated in its design and coordination and performed the statistical analysis. WL conceived of the study, participated in its design and coordination, performed the statistical analysis and helped to draft the manuscript. All authors of read and approved the manuscript.

## Pre-publication history

The pre-publication history for this paper can be accessed here:

http://www.biomedcentral.com/1471-2369/11/4/prepub
